# Confirmation of human ovulation in assisted reproduction using an adhesive axillary thermometer (femSense^®^)

**DOI:** 10.3389/fdgth.2022.930010

**Published:** 2022-09-19

**Authors:** Gregor Weiss, Karl Strohmayer, Werner Koele, Nina Reinschissler, Michael Schenk

**Affiliations:** ^1^ Das Kinderwunsch Institut Schenk GmbH, Dobl, Austria; ^2^ SteadySense GmbH, Seiersberg, Austria; ^3^ Medical University of Graz, Department of Obstetrics and Gynecology, Graz, Austria

**Keywords:** ovulation, fertile window, basal temperature, femSense, reproduction

## Abstract

**Objective:**

Timing for sexual intercourse is important in achieving pregnancy in natural menstrual cycles. Different methods of detecting the fertile window have been invented, among them luteinization hormone (LH) to predict ovulation and biphasic body basal temperature (BBT) to confirm ovulation retrospectively. The gold standard to detect ovulation in gynecology practice remains transvaginal ultrasonography in combination with serum progesterone. In this study we evaluated a wearable temperature sensing patch (femSense®) using continuous body temperature measurement to confirm ovulation and determine the end of the fertile window.

**Methods:**

96 participants received the femSense® system consisting of an adhesive axillary thermometer patch and a smartphone application, where patients were asked to document information about their previous 3 cycles. Based on the participants data, the app predicted the cycle length and the estimated day of ovulation. From these predictions, the most probable fertile window and the day for applying the patch were derived. Participants applied and activated the femSense® patch on the calculated date, from which the patch continuously recorded their body temperature throughout a period of up to 7 days to confirm ovulation. Patients documented their daily urinary LH test positivity, and a transvaginal ultrasound was performed on day cycle day 7, 10, 12 and 14/15 to investigate the growth of one dominant follicle. If a follicle reached 15 mm in diameter, an ultrasound examination was carried out every day consecutively until ovulation. On the day ovulation was detected, serum progesterone was measured to confirm the results of the ultrasound. The performance of femSense® was evaluated by comparing the day of ovulation confirmation with the results of ovulation prediction (LH test) and detection (transvaginal ultrasound).

**Results:**

The femSense® system confirmed ovulation occurrence in 60 cases (81.1%) compared to 48 predicted cases (64.9%) with the LH test (*p* = 0.041). Subgroup analysis revealed a positive trend for the femSense® system of specific ovulation confirmation within the fertile window of 24 h after ovulation in 42 of 74 cases (56.8%). Cycle length, therapy method or infertility reason of the patient did not influence accuracy of the femSense® system.

**Conclusions:**

The femSense® system poses a promising alternative to the traditional BBT method and is a valuable surrogate marker to transvaginal ultrasound for confirmation of ovulation.

## Introduction

Human reproduction is a complex process, tightly linked to a chronological timeframe. Human sperm cells survive up to six days in the female vaginal tract, whereas the oocyte can only be fertilized within 12–24 h after ovulation (1). The “fertile window”, when intercourse can result in pregnancy, comprises a time frame of 5 days before ovulation and the day of ovulation itself (2). Consequently, the timing for sexual intercourse plays a major role in achieving pregnancy in natural menstrual cycles.

Follicular stimulating hormone (FSH) induces the growth of follicles inside the female ovary and one dominant follicle is selected and induced to grow (3). The high level of estrogen produced by the dominant follicle induces the release of luteinization hormone (LH), which triggers the ovulation (4). The abrupt secretion of LH into the bloodstream is induced by serum estradiol (produced from the dominant follicle) through a positive feedback mechanism impacting the anterior pituary gland (5). With a mean duration of 3 days, the LH surge is defined as an abrupt onset resulting in a peak, followed by a gradual descent until baseline (6). 35–44 h prior to ovulation the LH surge starts and reaches its peak 10–12 h before ovulation (5). Recent literature provides evidence that LH peak is best described as a wave with different surge variants rather than a peak (7, 8). While measurement of serum LH levels is invasive and impractical, urinary LH levels have been proven accurate and are used as unexpensive way to detect the fertile window (9). The general recommendation to start LH testing with the best predictive value for ovulation within 24 h is day 7 of the cycle (8). Due to its easy handling, the LH surge measurement has become popular within the last years. Despite positive correlations between urinary LH tests and ovulation, LH surges are very variable in configuration, amplitude and duration (7). Furthermore, urinary LH assessments are mostly qualitative based on the respective threshold of the used test. Crossing this threshold (= test positivity), indicates the approaching ovulation and does not provide information about the onset of the rise and how long the level already persists (9).

Besides the LH surge, different methods of detecting the occurrence of ovulation have been invented. Among these, monitoring of basal body temperature (BBT), discovered in the early 1900s, has established itself as a simple and non-invasive method to confirm ovulation (9, 10). As the BBT is measured orally, vaginally or rectally, it is defined as the core temperature in a resting state immediately after wake up or before physical activity (11). During the menstrual cycle, the BBT changes due to hormonal alterations as rise of estrogen and reaches the lowest point (nadir) at the fertile window prior to ovulation. After ovulation occurs, a woman's BBT typically increases with the rise of progesterone (12, 13). This increase in BBT lies in the range of 0.2–0.5°C and lasts until the onset of menstruation (14). Due to those minimal changes in temperature, women need to use a good quality thermometer with the ability to measure accurately. There are specific smartphone devices, online services, and printable charts available for women to document their BBT. This method depends on the right handling and is prone to indication errors. Furthermore, the need to measure the temperature every day at the same time directly after waking up as well as the interpretation of the documented results requires a high level of user compliance (15). Hence, a convenient and easy to use method is warranted to overcome issues in ovulation confirmation.

In clinical practice transvaginal ultrasonography in combination with serum progesterone levels performed by experienced clinicians is still the gold standard to determine ovulation by observing the dominant follicle and its rupture. This method is mainly used in specialized clinics or in assisted reproductive technologies (ARTs) to overcome infertility problems. However, it is still a cost-intensive and time-consuming way and not reasonably practicable (16). Therefore, the aim of the present study was to evaluate the performance of a wearable temperature sensing patch (femSense®) using continuous body temperature measurement to confirm ovulation and determine the end of the fertile window. The system predicts the cycle length as well as day of ovulation from the historic cycle data of the user, suggests the fertile window and determines when a patch should be applied according to a system-based algorithm using statistical measures. The system then confirms ovulation by detecting the post-ovulatory rise in the body temperature data recorded by the patch. We hypothesized that the measurement of BBT with femSense® may be a valuable surrogate marker to ultrasound in confirming ovulation.

## Material and methods

### Study population

96 participants were recruited at the fertility clinic “Das Kinderwunsch Institut Schenk GmbH” (Dobl, Austria) between October 2019 and December 2020. Women were included in the study if the following inclusion criteria were met: (A) primary/secondary infertility, (B) follicle monitoring cycle or frozen embryo transfer (fET), (C) good and healthy condition, (D) age between 20 and 40, (E) BMI between 18.5 and 30 and (F) near-field-communication equipped smartphones. Patients with chronic diseases, nicotine (more than 5 cigarettes per day) and alcohol abuse (more than 16 g alcohol per day) were excluded from this study. A follicle monitoring of all patients was performed using transvaginal ultrasound sonography (GE Voluson E8 BT09 ultrasound machine, GE Healthcare Austria GmbH, Austria). In addition, serum progesterone concentrations were determined using electrochemiluminescence immunoassay (ECLIA) for quantitative determination (Cobas-e411 analyzer, Roche Diagnostics GmbH, Austria). The study was approved by the Ethical Committee of the Medical University of Graz, Austria (approval number: 31-497ex18/19).

### Study process

**Figure 1:**
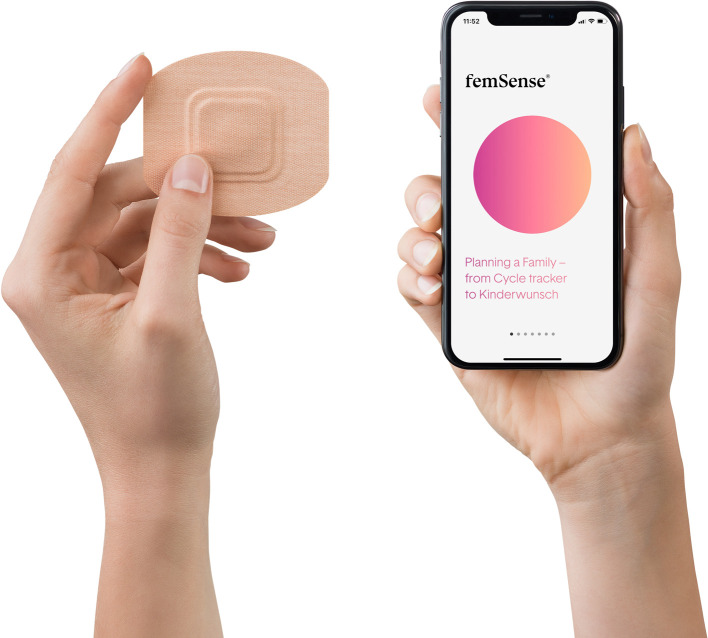
femSense^®^ system: The system consists of an adhesive axillary thermometer and a smartphone application (SteadySense GmbH, Austria).

After signing the informed consent, participants received the femSense® system consisting of an adhesive axillary thermometer, referred to as “patch”, and a smartphone application (SteadySense GmbH, Austria) (Figure 1). The patch is composed of a precise temperature sensor encased in biocompatible adhesive materials, the femSense® app acts as an interface between the patch and the user. Within the app, participants were asked to fill in data such as cycle length for the last 3 cycles and start date of their last menstrual cycle. According to the manufacturer, the reliability of cycle predictions is highest, when users enter at least 3 historic menstrual cycles as input data. Based on the cycle predictions, the patch was applied 4 days prior to the estimated day of ovulation to acquire sufficient amounts of temperature data and to account for variations between the participant's actual menstrual cycle length and the prediction.

The fertile window of a participant's current menstrual cycle was predicted by computing the cycle duration based on the available cycle data (cycle length and cycle variability) provided by the participant using statistical computation measures. Based on the cycle duration prediction, mathematical models estimated the most probable ovulation date, which was typically 10–14 days before the onset of menstruation, depending on the participant's individual cycle durations and their variability. The start of the fertile window was estimated to be 4 days before the predicted ovulation date, which was the date, where a patch could be applied. The end of the fertile window was estimated to be two days after the estimated ovulation date. If a patch confirmed ovulation based on the acquired temperature data, the end of the fertile window was set to one day after the confirmed day of ovulation.

Participants were asked to prepare the skin, apply, and start the patch according to the instructions for use. Afterwards, the patch recorded the temperature of the user every ten minutes. The recorded temperature information was stored within the memory of the patch and read out *via* the app using near-field-communication (NFC) technology. The temperature data was transmitted to servers, where algorithms analyzed the data. Once the algorithms detected the post-ovulatory temperature rise and confirmed ovulation, or after 7 days of wearing the patch, the participant was informed about the results and asked to remove the patch. While the patch was applied, patients were asked to measure and document their urinary LH test positivity every day at 02:30 *p*.m. with urinary LH tests (DocLab GmbH, Germany). A positive LH test was defined as a test result above a defined concentration threshold (25 mIU/ml).

Participants had transvaginal ultrasound on the 7th day of their cycle and on cycle day 10, 12 and 14/15 to investigate the growth of one dominant follicle. If a follicle reached 15 mm in diameter an ultrasound examination was carried out every day consecutively until ovulation. When ovulation was detected, serum progesterone was measured to confirm the results of the ultrasound. The performance of femSense® was evaluated by comparing the day of ovulation confirmation with the results of ovulation prediction (LH test) and detection (transvaginal ultrasound). Accordance of LH test and femSense® system in prediction and confirmation of ovulation was analyzed and compared to the actual day of ovulation detection with ultrasound.

For subgroup analysis, female cycles were divided into cycles with a length of 28 days, shorter cycles (<28 days) and longer cycles (>28 days). The therapies of the patients were divided into frozen embryo transfer (fET), intrauterine insemination (IUI) and cycle monitoring for timed intercourse (TI). Furthermore, participants were divided according to their infertility reasons into female factor (polycystic ovary syndrome, endometriosis, tubal factor), unexplained infertility, or male factor infertility.

### Calculating the day of ovulation:

**Ultrasound and serum progesterone**: Transvaginal ultrasound was used to detect the dominant follicle and ovulation. Using serial ultrasonography examinations, the time of ovulation was determined as the point between maximum follicular diameter and follicular collapse. Signs of ovulation include disappearance or decrease of follicle size, increased echogenicity inside the follicle (indicating corpus luteum formation) and free fluid in pelvis (9). If ovulation was detected serum progesterone levels were analyzed to further confirm the result of the ultrasound.

**Urinary LH test**: The rise in urine LH levels is known to occur near the time when ovulation takes place during the menstrual cycle (8). The test shows whether the cut-off value has been exceeded and ovulation has been predicted. The time when the urinary test showed a positive result (above the defined concentration threshold) was annotated as time for ovulation.

**femSense**® **system**: An algorithm within the femSense app predicted the fertile window based on the cycle data entered by the participant. Based on this, the date to apply a patch for continuous temperature measurement throughout the predicted fertile window was determined. After being applied and activated, the femSense® patch continuously recorded axillary body temperature data. With each read-out by the participant the recorded temperature data series was analyzed by the ovulation algorithm to detect the post-ovulatory rise in body temperature in order to retrospectively confirm that ovulation had occurred. Upon detection, the algorithm tracked the origin of the temperature rise and the date, where it originated, marked the day of ovulation that was displayed to the participant. Depending on the timing of the readout *via* NFC, the day of ovulation was usually the day before, or the day where the post-ovulatory temperature rise was detected by the femSense® ovulation algorithm.

### Statistical analysis

Associations between nominal variables were computed by Chi-Squared/Fisher's exact test depending on the number of included cases. A *p*-value (one-tailed) of <0.05 was considered as statistically significant. Statistical analysis was performed using SPSS 23.0.0.2 (SPSS Inc., Chicago, USA) for calculations as well as Microsoft Excel (Microsoft, USA) as support for visualizations.

## Results

### Overall detection and confirmation of the ovulation process

**Figure 2:**
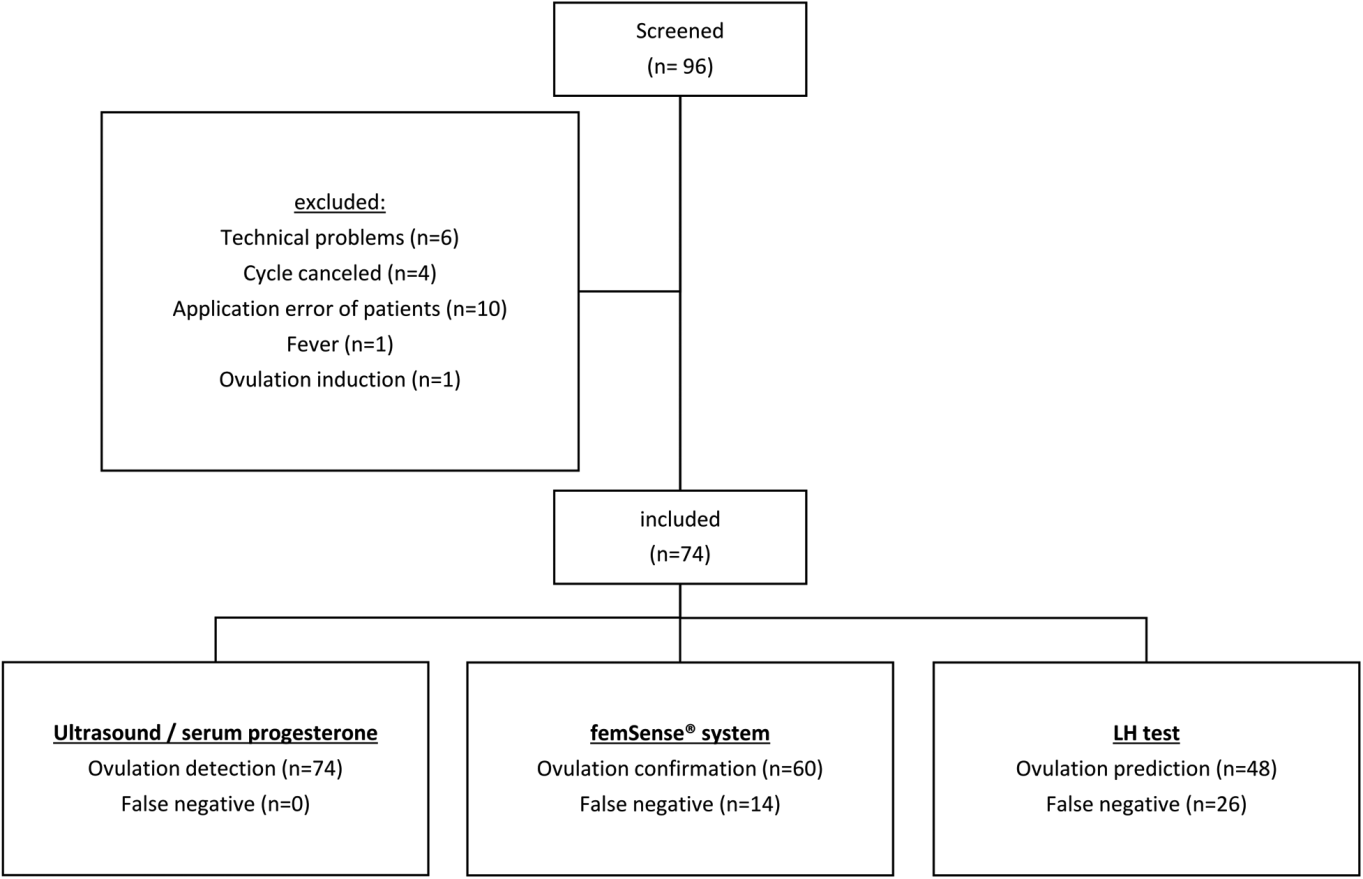
Study population flow chart: after screening 22 out of 96 participants were excluded. Ultrasound together with serum progesterone detected 74 ovulations, the femSense^®^ system confirmed 60 and the urinary LH test predicted 48 ovulations, respectively.

**Figure 3:**
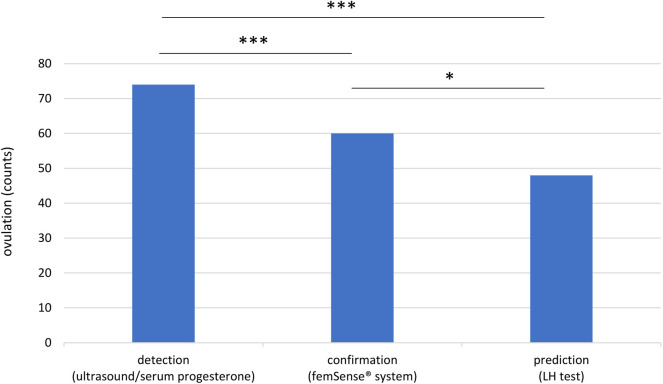
Overview of ovulation detection, confirmation, and prediction: the femSense^®^ system confirmed the ovulation in 60 participants while the LH test predicted ovulation in 48 participants. Significant differences are indicated as follows: *p < 0.05; ***p < 0.001.

Out of 96 recruited participants 74 (77.1%) were included in the trial. 22 participants (22.9%) were excluded due to technical problems (6 technical problems with the femSense® system) or other reasons (4 cycle cancellations due to patients wish, 10 improper usage events, 1 fever event, 1 induced ovulation). Using ultrasound and serum progesterone measurement (gold standard) ovulation was detected in all 74 cases. The femSense® system confirmed the ovulation in 60 cases (81.1%) and failed confirmation in 14 cases (18.9%) compared to ultrasound. The LH test predicted 48 ovulations (64.9%) and failed prediction in 26 patients (35.1%) (Figure 2). The femSense® system confirmed more ovulations than the LH test was able to predict (*p* = 0.041) (Figure 3).

### Method accordance and subgroup analysis

**Figure 4:**
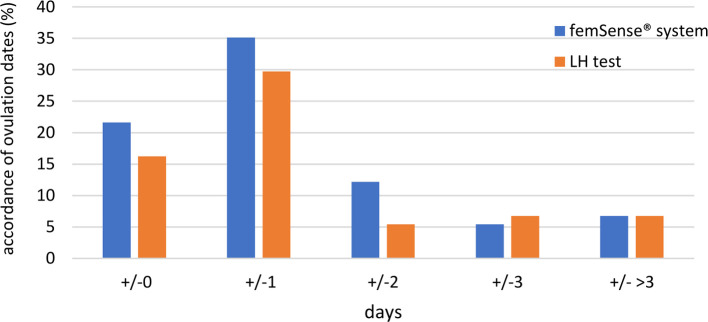
Accordance of ovulation days of femSense^®^ system and LH test compared to ultrasound: An exact accordance of the patch was found in 16 participants (21.7%), a difference of one day in 26 participants (35.1%), a difference of two days in 9 participants (12.2%), a difference of three days in 4 participants (5.4%) and a difference of more than three days in 5 participants (6.8%). An exact accordance of the LH test was found in 12 participants (16.2%), a difference of one day in 22 participants (29.7%), a difference of two days in 4 participants (5.4%), a difference of three days in 5 participants (6.8%) and a difference of more than three days in 5 participants (6.8%).

The femSense® system confirmed ultrasound measurement in 16 participants (21.7%) with exact accordance. The difference between the femSense® system and ultrasound in confirming day of ovulation occurrence of one day was found in 26 participants (35.1%), two days in 9 participants (12.2%), three days in 4 participants (5.4%) and more than three days in 5 participants (6.8%). Ovulation prediction of the LH test was consistent with the ultrasound detection in 12 participants (16.2%). The difference between LH tests and ultrasound of one day was found in 22 participants (29.7%), two days in 4 participants (5.4%), three days in 5 participants (6.8%) and more than three days in 5 participants (6.8%), see (Figure 4).

**Figure 5:**
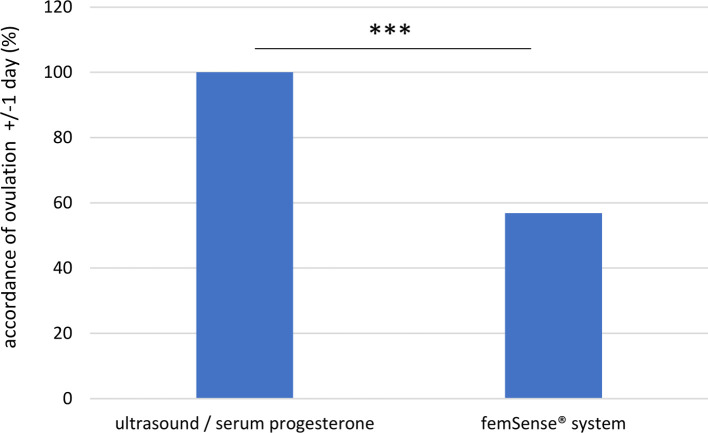
Exact accordance of ovulation confirmation (±/− 1 day): the femSense^®^ system confirmed the correct ovulation day in 42 (56.8%) of all participants. Significant differences are indicated as follows: ***p < 0.001.

As the oocyte is only able to be fertilized between 12 and 24 h after ovulation, ovulation days of the femSense® system with a difference to the transvaginal ultrasound detection of +/−1 day were investigated in more detail. As a result, the femSense® system confirmed the day of ovulation occurrence in 42 of 74 cases (56.8%) (Figure 5).

Subgroup analysis revealed no changes in accuracy of the different methods for ovulation prediction, detection and confirmation depending on cycle length (28 days, <28 days, >28 days), therapy method (fET, IUI, TI) or infertility reason (female factor, unexplained infertility, or male factor infertility) (Supplementary Table S1).

## Discussion

In the present study we were able to demonstrate that continuously measured body temperature with the femSense® system confirmed the process of ovulation in 81.1% of the participants. Furthermore, subgroup analysis revealed a positive trend for the femSense® system of specific ovulation confirmation within the important timeframe of 24 h after ovulation. This study is the first to compare the ovulation confirmation of the temperature based femSense® system with the LH surge method and transvaginal ultrasound.

Since infertility is an ever-increasing global phenomenon, affecting between 8% and 12% of all the couples worldwide, detecting the fertile window is one of the major goals in family planning. BBT has been used for decades to help women optimize the timing of intercourse during a fertile window even though it is influenced by environmental factors like inflammation, fever, emotional stressors or alcohol consumption (9, 17). Tracking the ovulatory cycles by measuring the oral temperature after awakening has been proven a popular method since it is easy to use and noninvasive (12). However, these daily point temperature evaluations are sensitive to the time of measurement and shifts can easily go undetected (18). The reliability of BBT measured rectally in the morning to detect the ovulation was investigated by de Mouzon et al. Because of the large number of false nadirs and temperature rises and the difficulty in interpreting BBT charts they concluded the BBT method as an unreliable indicator of ovulation (19). Numerous studies have questioned the reliability of BBT and report an accuracy of approximately 22% in confirming ovulation (9, 18). However, new technologies in temperature sensing and a better understanding of ovulation physiology bring back BBT to the spotlight in recent studies. In this context, tracking of daily activities including body temperature changes over 24 h, personalized self-monitoring devices, like vaginal thermometer sensors, in-ear thermometers or wrist wearable thermometers were developed (9, 16, 20). Écochard and coworkers described the relationship between BBT and pregnanediol-3 alpha-glucuronide (PDG, the urine metabolite of progesterone) and demonstrated the high reliability of BBT to confirm ovulation (21). In line with these findings, we were able to demonstrate the tremendous potential of continuous temperature measurement for confirmation of ovulation in more than 80% of the participants using the femSense® system.

The skin temperature is generally lower than the BBT because they are not close to the major blood vessels and exposed to the environment. The difference is not a fixed amount and the heat transfer from the core to the surface does not occur immediately (22). One of the advantages of using the femSense® system compared to oral, vaginal, or rectal BBT method is the continuous measurement of the temperature in ten-minute intervals, providing the user with a timely independence in terms of data acquisition. Hence, the risks of errors due to different wakeup times, missing measurements or different measurement positions are minimized. In contrast to the traditional BBT temperature method, users are not limited to measuring at exact times and there is no need to enter or write down and analyze the manually measured temperature values. However, some participants had technical difficulties in performing the patch-interactions with the smartphone application through the NFC-interface, which can be partially accounted to the unfamiliarity of participants with this technology. Additionally, it is necessary for the femSense® system that the user reads out the patch on a regular basis, ideally twice a day, which both pose potential error sources and may be one of the reasons for the exclusion rate.

When comparing LH tests and BBT it's necessary to keep in mind that these methods complement each other in detecting the fertile window. A rise in LH occurs near the time of ovulation, while BBT rises as a consequence thereof. Interestingly, recent literature findings provide evidence that LH testing to predict ovulation may be affected negatively by several confounding factors like timing of the test during the cycle, quality of the lateral flow assay, ease of interpretation by the participant, threshold of the test and the variability of LH secretion in individual women (8).

In our study, the ovulation prediction using the LH test revealed more than 33% false negative results, indicating user compliance issues, insufficient hormone changes throughout the ovulation, or impaired LH stripes (23). In comparison, the femSense® system failed confirmation of ovulation in approximately 19%, demonstrating a better performance and a higher sensitivity than the LH test. Compared to the ultrasound method, femSense® revealed a remarkable high ovulation confirmation rate. However, the precision in terms of accurately confirming the day of ovulation admits of improvement. For slightly more than half of the investigated cycles, the confirmation of femSense® correlated with the ultrasound detection within +/−1 day. This result may be explained by the fact, that the postovulatory temperature rise is not an immediate event. Écochard recently demonstrated an increase in BBT (and PDG) two to three days, on average, before ovulation itself (21). Hence, the rate of BBT increase may differ between patients and cause a delay in the detection of the postovulatory temperature rise and confirmation of ovulation. In addition, the false negative rate of the femSense® system shows that ovulation could not be confirmed in every 5th investigated cycle, which can be accounted to the individual nature of body temperature and the significance of the postovulatory temperature rise, which can differ from user to user.

The high rate of false negative results of LH tests in our study may be explained with different LH surge variants. Direito et al. described the LH surge variants (short, medium, double, prolonged surge, single peak, plateau, double peak, multiple peaks) in ovulating women and reported extreme variability in LH surges in terms of amplitude and duration. They correlated multiple peak LH surges with smaller preovulatory follicles and prolonged LH surges (more than 3 days after ovulation) with delayed luteinization, indicating possible luteal insufficiency (7). In addition, the LH surge was discovered to be a better marker than the LH peak itself to predict ovulation (24). As the ovulation and possibility of conception often occurs prior to the detectable LH surge, LH tests only indicate half of all ovulations. In participants with higher basal LH levels and polycystic ovary syndromes, LH tests often detect false positive ovulations (25). These results underline the complex LH physiology which needs to be taken into account when interpreting LH test results. Hence, LH may serve as complementary marker but is not recommended to be used solely to define the end of the fertile window.

In the present study we were able to demonstrate that the femSense® system performs reliably independent of the individual participant's medical background. The system revealed no changes in accuracy of ovulation confirmation depending on cycle length (28 days, <28 days, >28 days), therapy method (fET, IUI, TI) or infertility reason (female factor, unexplained infertility, or male factor infertility), indicating the reliability of the system independent of the individual participant background. With the investigated system, ovulation was identified in over 80% of all observed cycles, which is comparable with the results of previous studies examining BBT. However, the femSense® system did not capture and confirm all ovulations.

## Conclusion

The femSense® system provides a reliable, user-friendly, and non-invasive method to confirm ovulation by measuring hormone-induced temperature changes. Moreover, the system adapts to the lifestyle of the woman using it. However, the usability might be increased by simplifying the connection of the patch with the smartphone application. The femSense® system poses a promising alternative to the traditional BBT method and is a valuable surrogate marker to transvaginal ultrasound for confirmation of ovulation.

## Data Availability

The raw data supporting the conclusions of this article will be made available by the authors, without undue reservation.
