# Supplementary material for: Confirmation of human ovulation in assisted reproduction using an adhesive axillary thermometer (femSense®)
Source: Front Digit Health. 2022 Sep 19;4:930010. doi: 10.3389/fdgth.2022.930010 (PMC9634753; doi:10.3389/fdgth.2022.930010)
Supplement: Supplementary file 1 [file Table1.docx]

|  | Accordance | **Cycle length** | | | **therapy method** | | | **infertility reason** | | |
| --- | --- | --- | --- | --- | --- | --- | --- | --- | --- | --- |
|  |  | *28d* | *>28d* | *<28d* | *fET* | *IUI* | *TI* | *male factor* | *unexplained* | *female factor* |
| femSense^®^ | +/-0 | 14 | 1 | 1 | 12 | 4 | 0 | 7 | 4 | 5 |
|  | +/-1 | 19 | 5 | 2 | 21 | 4 | 1 | 11 | 3 | 12 |
|  | +/-2 | 6 | 1 | 2 | 7 | 1 | 1 | 0 | 2 | 7 |
|  | +/-3 | 3 | 1 | 0 | 3 | 0 | 1 | 4 | 0 | 0 |
|  | +/- >3 | 4 | 1 | 0 | 5 | 0 | 0 | 2 | 1 | 2 |
|  | false negative | 10 | 1 | 3 | 10 | 3 | 1 | 6 | 4 | 4 |
| LH test | +/-0 | 9 | 3 | 0 | 8 | 2 | 2 | 4 | 1 | 6 |
|  | +/-1 | 18 | 1 | 3 | 16 | 6 | 0 | 8 | 4 | 6 |
|  | +/-2 | 4 | 0 | 0 | 4 | 0 | 0 | 2 | 0 | 1 |
|  | +/-3 | 3 | 1 | 1 | 4 | 0 | 1 | 3 | 1 | 0 |
|  | +/- >3 | 5 | 0 | 0 | 4 | 0 | 1 | 3 | 0 | 1 |
|  | false negative | 17 | 5 | 4 | 22 | 4 | 0 | 10 | 8 | 16 |
| *d (days); fET (frozen embryo transfer); IUI (intrauterine insemination), TI (timed intercourse); female factor (polycystic ovarian syndrome, endometriosis, tubal factor)* | | | | | | | | | | |

**Supplementary Table 1**: Accordance of the femSense^®^ system and the LH test in relation to cycle length, therapy method and infertility reason.
